# Transcriptomics unravels molecular players shaping dorsal lip hypertrophy in the vacuum cleaner cichlid, *Gnathochromis permaxillaris*

**DOI:** 10.1186/s12864-021-07775-z

**Published:** 2021-07-05

**Authors:** Laurène Alicia Lecaudey, Pooja Singh, Christian Sturmbauer, Anna Duenser, Wolfgang Gessl, Ehsan Pashay Ahi

**Affiliations:** 1 Institute of Biology, University of Graz, Universitätsplatz 2, A-8010 Graz, Austria; 2 Department of Natural History, NTNU University Museum, Norwegian University of Science and Technology, NO-7491 Trondheim, Norway; 3 Department of Biological Sciences, University of Calgary, 2500 University Dr NW, Calgary, AB T2N 1N4 Canada; 4 Organismal and Evolutionary Biology Research Programme, University of Helsinki, Viikinkaari 9, 00014 Helsinki, Finland

**Keywords:** Lip hypertrophy, Gene expression, Cichlidae, Adaptation, RNA-seq, Lake Tanganyika

## Abstract

**Background:**

Teleosts display a spectacular diversity of craniofacial adaptations that often mediates ecological specializations. A considerable amount of research has revealed molecular players underlying skeletal craniofacial morphologies, but less is known about soft craniofacial phenotypes. Here we focus on an example of lip hypertrophy in the benthivorous Lake Tangnayika cichlid, *Gnathochromis permaxillaris*, considered to be a morphological adaptation to extract invertebrates out of the uppermost layer of mud bottom. We investigate the molecular and regulatory basis of lip hypertrophy in *G. permaxillaris* using a comparative transcriptomic approach.

**Results:**

We identified a gene regulatory network involved in tissue overgrowth and cellular hypertrophy, potentially associated with the formation of a locally restricted hypertrophic lip in a teleost fish species. Of particular interest were the increased expression level of *apoda* and *fhl2*, as well as reduced expression of *cyp1a*, *gimap8*, *lama5* and *rasal3*, in the hypertrophic lip region which have been implicated in lip formation in other vertebrates. Among the predicted upstream transcription factors, we found reduced expression of *foxp1* in the hypertrophic lip region, which is known to act as repressor of cell growth and proliferation, and its function has been associated with hypertrophy of upper lip in human.

**Conclusion:**

Our results provide a genetic foundation for future studies of molecular players shaping soft and exaggerated, but locally restricted, craniofacial morphological changes in fish and perhaps across vertebrates. In the future, we advocate integrating gene regulatory networks of various craniofacial phenotypes to understand how they collectively govern trophic and behavioural adaptations.

**Supplementary Information:**

The online version contains supplementary material available at 10.1186/s12864-021-07775-z.

## Introduction

Teleost fishes show striking adaptive diversity in their craniofacial anatomy, which reflects an equally striking variety of ecological and trophic specializations. It is therefore not surprising that this phenotypic diversity and its adaptive background has attracted the attention of developmental, molecular and evolutionary biologists, beyond model species such as zebrafish and medaka [1, 2]. In the last two decades, an impressive set of molecular players participating in the development and morphogenesis of craniofacial skeletal structures, including their interconnecting signalling pathways, have been described in teleost fishes [2–5]. Much less is known about the morphogenic molecular players and underlying signals of craniofacial soft tissues, that also exhibit varied adaptive peculiarities. In cichlid fishes, a novel model system for adaptive radiation, the molecular mechanisms underlying exaggerated soft-tissue morphologies in lip, nose and nuchal hump phenotypes have been addressed only recently [6–11].

One such exaggerated craniofacial soft-tissue phenotype is the overgrowth of the lip tissue, the so-called thick-lipped phenotype, which has been observed in cichlid species in lakes spanning different continents [7, 8, 12]. The repeated evolution of the hypertrophic lip phenotype, in both African and Central American cichlids, has been associated with the dietary adaptation to suck elusive invertebrates out of narrow crevices in rocky habitats [8, 13, 14]. Genetic studies so far have suggested numerous loci across the genome whose small additive effects can be linked to a variety of thick-lipped phenotypes [7, 8, 12]. These findings indicate that the thick-lipped phenotype is a complex trait [12]. The exaggerated thickening of the lip might not follow a uniform pattern across the entire upper and lower lip and the extent of lip hypertrophy is significantly reduced in captivity under flake food diet, when the fish are not using this foraging strategy, so that lip hypertrophy seems highly dependent on foraging performance, making it a phenotypically plastic trait [12]. Both genetic and phenotypic plasticity take part in the adaptive morphogenesis of this phenotype [6, 15].

**Fig. 1:**
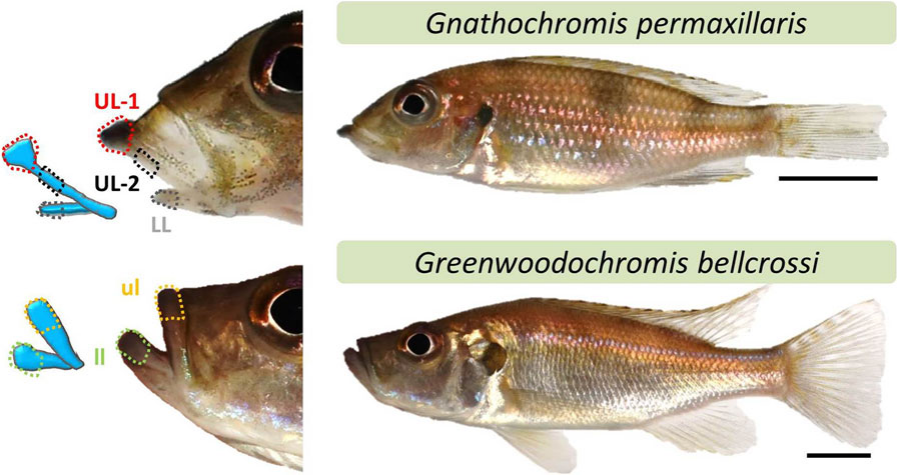
Two East African cichlid species of the tribe Limnochromini from Lake Tanganyika used in this study. The areas from which the soft tissue samples are taken in lips are delineated by coloured dash lines; UL/ul and LL/ll refer to upper and lower lip, respectively


Another interesting example of lip hypertrophy is found in the Lake Tanganyika cichlid adaptive radiation, in the species *Gnathochromis permaxillari****s***. It has a shovel-like snout with a peculiarly thickened part of the upper lip [16]. *G. permaxillaris* is a benthivorous deepwater cichlid of the tribe Limnochromini [16] that develops a unique hypertrophy in the most anterior part of its upper lip (Fig. 1). The species slowly swims directly over the mud surface with its protruding snout hovering at the surface and at the same time opens the protruding mouth underneath, while ingesting mud that is then filtered through the gill arches. Its common name ‘the Vacuum cleaner cichlid’ is an adequate description of this feeding mode. Thereby, the protruding upper lip with its small hypertrophy at its tip seems to be adaptive by boosting the efficiency of filtration (unpublished behavioural observations by Heinz H. Bücher; see Video S1). Interestingly, when fish are raised in captivity, individuals develop this phenotype to a lesser extent (Peter Henninger, personal communication), again suggesting some degree of phenotypic plasticity. Nothing is known so far about the molecular basis of lip morphogenesis in *G. permaxillaris*.

**Additional file 3: Video S1.** Foraging behaviour of the vacuum cleaner cichlid in its natural habitat.

In this study, we aimed to identify genes playing a role in the formation of the dorsal part of the upper lip, using RNA-sequencing. To this end, we profiled gene expression differences between the hypertrophic part of the upper lip versus the posterior part of the upper lip and the most anterior part of the lower lip, the latter two do not exhibit hypertrophic overgrowth in wild-caught young male adults of *G. permaxillaris* (Fig. 1). In order to add another layer of filtering, we also conducted an inter-specific comparison using a closely related species in the tribe Limnochromini, *Greenwoodochromis bellcrossi* [16–18], which does not have a protruding upper lip but displays hypertrophy in the entire parts of both the upper and lower lips (Fig. 1). Using the regulatory sequences of differentially expressed genes in the hypertrophic lip region, we predicted their potential upstream transcriptional regulators, and applied qPCR expression analysis on the most interesting candidate genes to validate our results from the RNA-seq analysis. We identified a gene regulatory network, potentially associated with the formation of a locally restricted hypertrophic lip in the Limnochromini. Our results provide a foundation for future investigations of molecular players shaping exaggerated but locally restricted morphological changes in facial elements of fish and vertebrates. Moreover, these findings can be further used for molecular comparisons between wild and captive bred *G. permaxillaris* in order to investigate the mechanisms underlying the plasticity of the lip hypertrophy in fish. Recent attempts have been made to unravel molecular factors underlying plastic responses to different mechanical stimuli in cichlid jaw skeleton [19–21], but studies focusing on craniofacial soft tissues are lacking. Our results pave the way for interesting future functional gene characterisation as well.

## Results

### RNA-seq, differential gene expression and downstream analyses

**Fig. 2:**
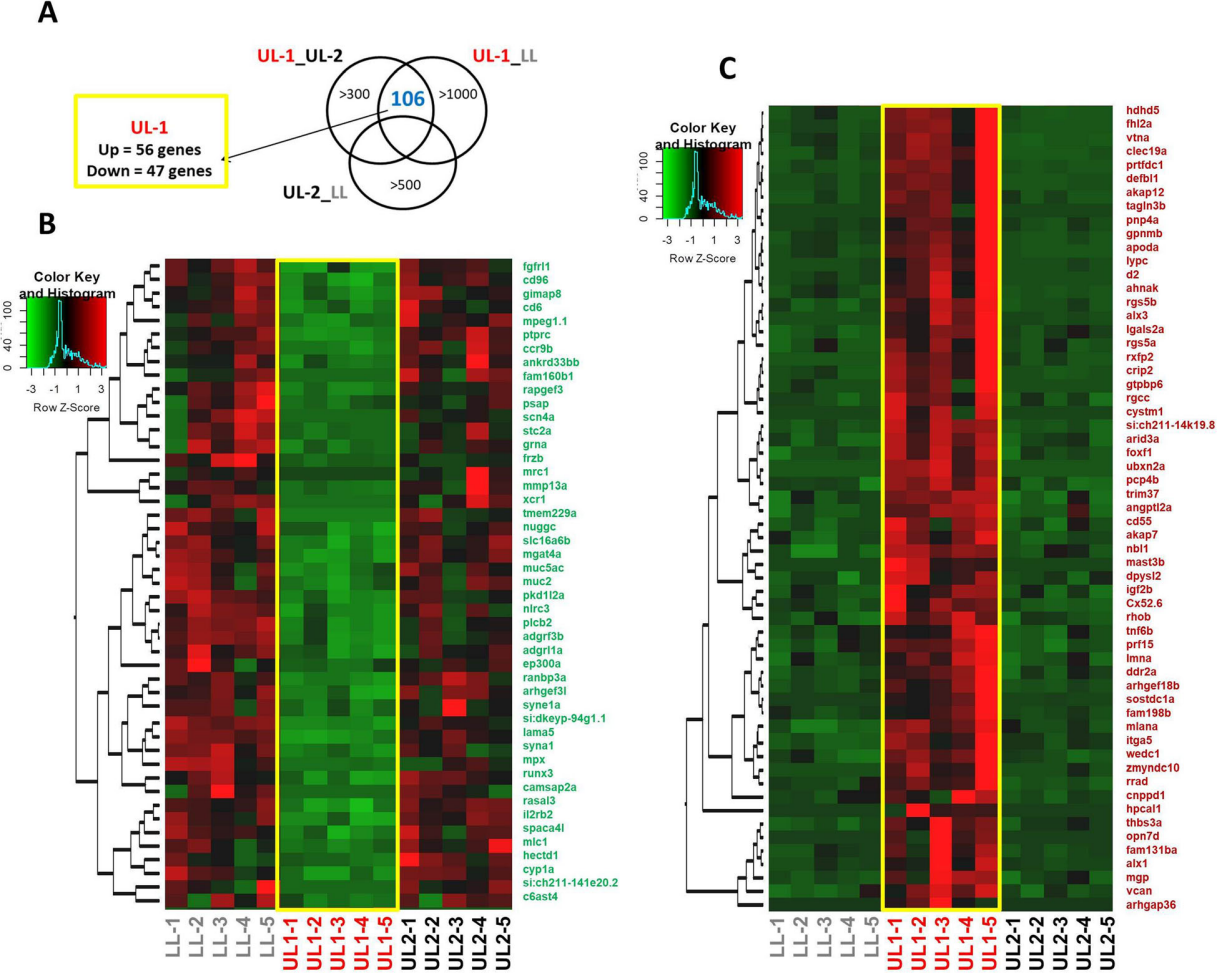
Differentially expressed genes in the hypertrophic region of dorsal lip in *Gnathochromis permaxillaris*. **A** Venn diagram representing 106 genes showing differential expression between the hypertrophic region. (UL-1) and other regions of the lip. Dendrogram clusters of genes with lower (**B**) and higher (**C**) expression in UL-1 region in *G. permaxillaris* compared to the other lip regions. Red and green shadings indicate higher and lower relative expression, respectively

The total RNA sequencing resulted between 3.84 and 8.13 million reads per sample and after removal of low quality reads, each sample had between 3.82 and 8.10 million reads (Table S1). The low reads for some of the samples limit the results of our study to differentially expressed (DE) genes that have relatively high expression levels, in other words, it is likely that some of the low expressed genes are not detected among the list of DE genes due to the low coverage of some samples. This can be particularly the case for comparisons involving *G. bellcrossi* since the two samples with less than 4 million reads are from this species. The raw sequence reads have been submitted to the Sequencing Read Archive (SRA) of NCBI (accession number: PRJNA694145). The comparisons of the lip regions of *G. permaxillaris* resulted in 106 DE genes between the hypertrophic part of the upper lip (UL-1) versus both the non-hypertrophic part of the upper lip (UL-2) and the lower lip (LL) (Fig. 2A). Among these, 56 genes showed increased expression, whereas 47 genes had reduced expression in UL-1 (Fig. 2B and C). The heatmap clustering of the DE genes revealed two major branches in the group of down-regulated genes (Fig. 2B), whereas only one major branch is observed in the group of up-regulated genes (Fig. 2C).

**Fig. 3:**
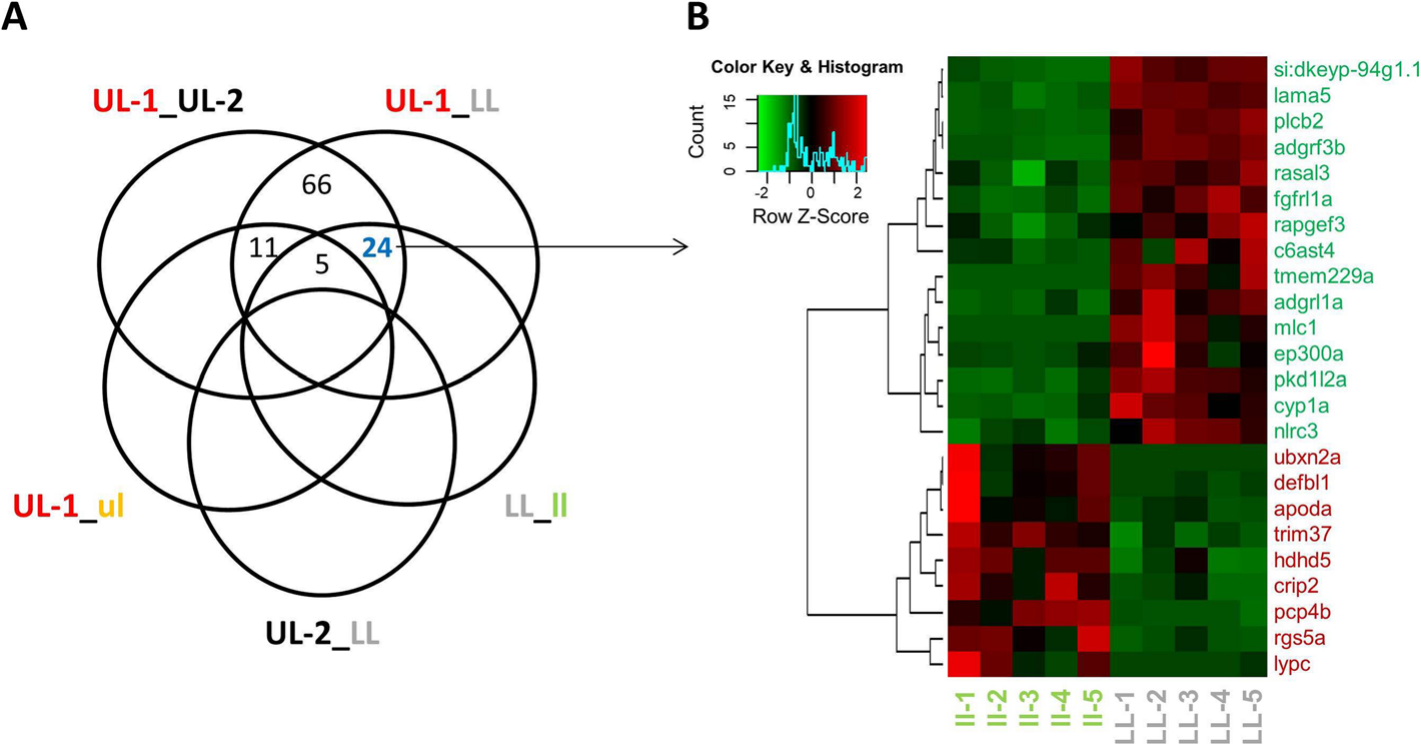
Differentially expressed genes in the hypertrophic region of the lips shared between *G. permaxillaris* and *G. bellcrossi*. **A**. Venn diagram representing 24 genes showing differential expression between the hypertrophic and non-hypertrophic lip regions between the species. **B**. Dendrogram clusters of the 24 shared DE genes in LL region in *G. permaxillaris* compared to the ll lip region in *G. bellcrossi*. Red and green shadings indicate higher and lower relative expression, respectively

We also conducted a second filtering step in order to check whether among the identified list of 106 genes above, any might play roles in lip hypertrophy in another closely related species. To do this, we first compared UL-1 from *G. permaxillaris* to the upper lip (ul) of *G. bellcrossi,* and since both regions have hypertrophy the DE genes between them were filtered out. Next, we compared LL from *G. permaxillaris* to the lower lip (ll) of *G. bellcrossi,* and this time, since ll has hypertrophy but not LL, we checked for genes which were shared with the list of 106 genes (from the within species comparisons). We only found 24 DE genes to be shared between the intra- and inter-specific comparisons, suggesting their potential roles in lip hypertrophy across the species (Fig. 3). Out of these, 9 genes showed higher and 15 showed lower expression in the hypertrophic lip region between the species (ll vesus LL), and similar expression patterns were observed for the within-species comparisons. Among these 24 genes, at least 5 genes, *apoda*, *cyp1a*, *lama5*, *rasal3* and *trim37*, have been already found to be involved in lip morphogenesis in other vertebrates (Table 3).

**Fig. 4:**
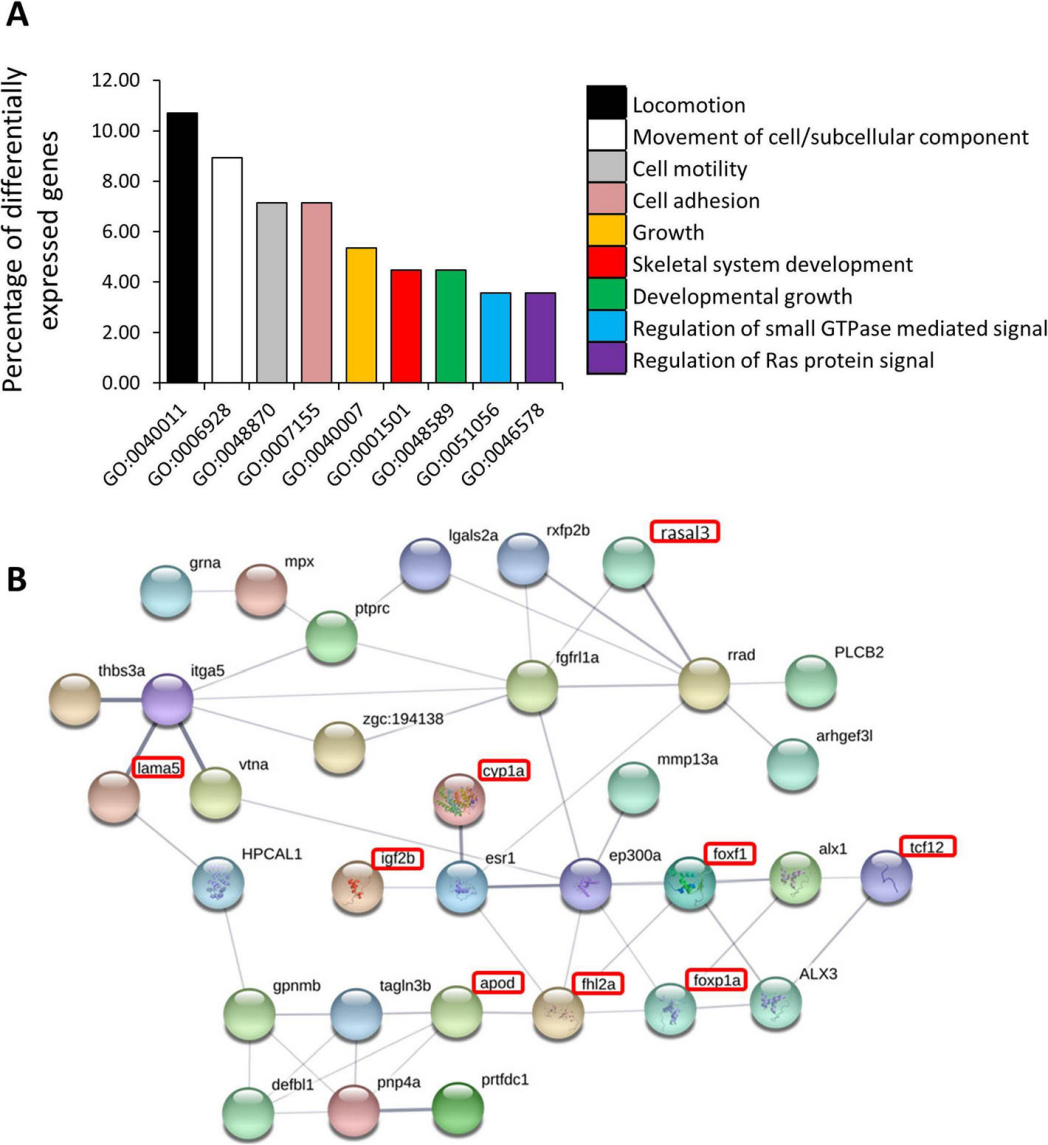
Downstream functional analyses of differentially expressed genes. **A**. Gene ontology enrichment analysis for biological processes, using the shared 106 differentially expressed genes, conducted with DAVID tool. **B**. Predicted functional associations/interactions between the differentially expressed genes based on zebrafish databases in STRING v10 (http://string-db.org/). The differential expression of the genes specified with red rectangles are confirmed by both qPCR and RNA-seq

Using the list of 106 DE genes, we conducted a gene ontology enrichment analysis, and amongst the significantly enriched GO terms, biological processes related to cell movement, adhesion and growth had the highest enrichment ratios (Fig. 4A). The enrichment for genes related to locomotion raises the possibility of functional specificity for the hypertrophy of the most anterior part of the upper lip of *G. permaxillaris*. We also found enrichments of molecular processes related to GTPase and Ras protein signalling for the list of DE genes. Subjecting the same gene list to an interactome analysis revealed only a single large interconnected network of genes. However, the network contained several interesting genes, and particularly transcription factors (TFs), with potential role in tissue overgrowth and cellular hypertrophy (Fig. 4B). We also performed overrepresentation analysis of TF binding motifs on the regulatory sequences of the DE genes using the MEME algorithm [22]. This resulted in five enriched motifs present in the regulatory sequences of at least 30 out of 106 DE genes (Table 1). In search for similarities of the identified motifs with known TF binding sites in vertebrates, we found at least two interesting TF candidates, *foxp1* and *heb/tcf12*, with overrepresented binding sites. Furthermore, both of these TFs showed regulatory connections with some of the DE genes in the identified interactome network, with *foxp1* having more connections than *tcf12* (Fig. 4B).

**Table 1:** Predicted binding sites for potential upstream regulators of the differentially expressed genes identified by RNA-seq. PWM ID indicates positional weight matrix ID of a predicted binding site and *E*-values refer to matching similarity between the predicted motif sequences and the PWM IDs. The count implies on number of genes containing the predicted motif sequence on their regulatory region

TF binding site	PWM ID	Count	Predicted motif sequence	***E***-value
HEB	M00698	97 / 106	CCTGCTG	1.119e-09
FOXP1	M00987	81 / 106	AAATAAANAACAAAAAAAAWA	4.441e-16
SMAD3	M00701	72 / 106	ACASASASACASACA	2.28E-07
HEB	M00698	41 / 106	KCCMRGCTGVCTGS	3.51E-07
FOXP1	M00987	31 / 106	TWTWYDTATWWRTWTATTTATWTATWWAT	1.05E-09
FOX	M00809			3.061E-08

### Expression validation using qPCR

The gene expression analysis by qPCR requires the identification of stably expressed reference genes [23], and our previous studies on East African cichlids, confirmed that the validation of suitable reference gene(s) is an essential step that needs to be taken in every species, every tissue and experimental condition [24–28]. In order to choose adequate reference gene candidates, we conducted a ranking for the genes with no expression difference (FDR = 1 in the RNA-seq comparisons) based on their coefficient variation (CV) throughout all the samples. The ten genes with lowest CV were selected as candidate reference genes for validation of their expression stability by qPCR. None of the validated reference genes in previous gene expression studies of East African cichlids have appeared among the ten candidates, confirming the necessity of reference gene validation for each experimental setup [24–29]. Overall, the reference gene rankings by the three algorithms, BestKeeper, geNorm and NormFinder software showed that two of the candidates, *pip4k2b* and *ss18,* were always among the top three most stable reference genes (Table 2). Therefore, we used geometric mean of Cq values for *pip4k2b* and *ss18* in each sample, as a normalization factor, in order to calculate the relative gene expression levels of our target genes.

**Table 2:** Ranking and statistical analyses of reference genes in the lip samples using three different algorithms

BestKeeper				geNorm		NormFinder	
Ranking	SD	Ranking	r	Ranking	M	Ranking	SV
*luc7l*	0.260	*ubxn4*	0.908	*pip4k2b*	0.328	*ss18*	0.193
*ss18*	0.289	*ss18*	0.905	*trir*	0.333	*pip4k2b*	0.242
*pip4k2b*	0.293	*pip4k2b*	0.902	*ss18*	0.335	*trir*	0.248
*trir*	0.296	*setd3*	0.878	*prkci*	0.341	*setd3*	0.258
*h3f3*	0.328	*trir*	0.878	*h3f3*	0.352	*h3f3*	0.274
*prkci*	0.366	*prkci*	0.873	*setd3*	0.356	*prkci*	0.303
*snrnp70*	0.391	*h3f3*	0.828	*ubxn4*	0.364	*ubxn4*	0.329
*vasp*	0.402	*vasp*	0.806	*vasp*	0.421	*luc7l*	0.333
*ubxn4*	0.402	*luc7l*	0.611	*luc7l*	0.425	*vasp*	0.411
*setd3*	0.414	*snrnp70*	0.389	*snrnp70*	0.590	*snrnp70*	0.445

Abbreviations: *SD* Standard deviation, *r* Pearson product-moment correlation coefficient, *SV* stability value, *M* M value of stability

**Fig. 5:**
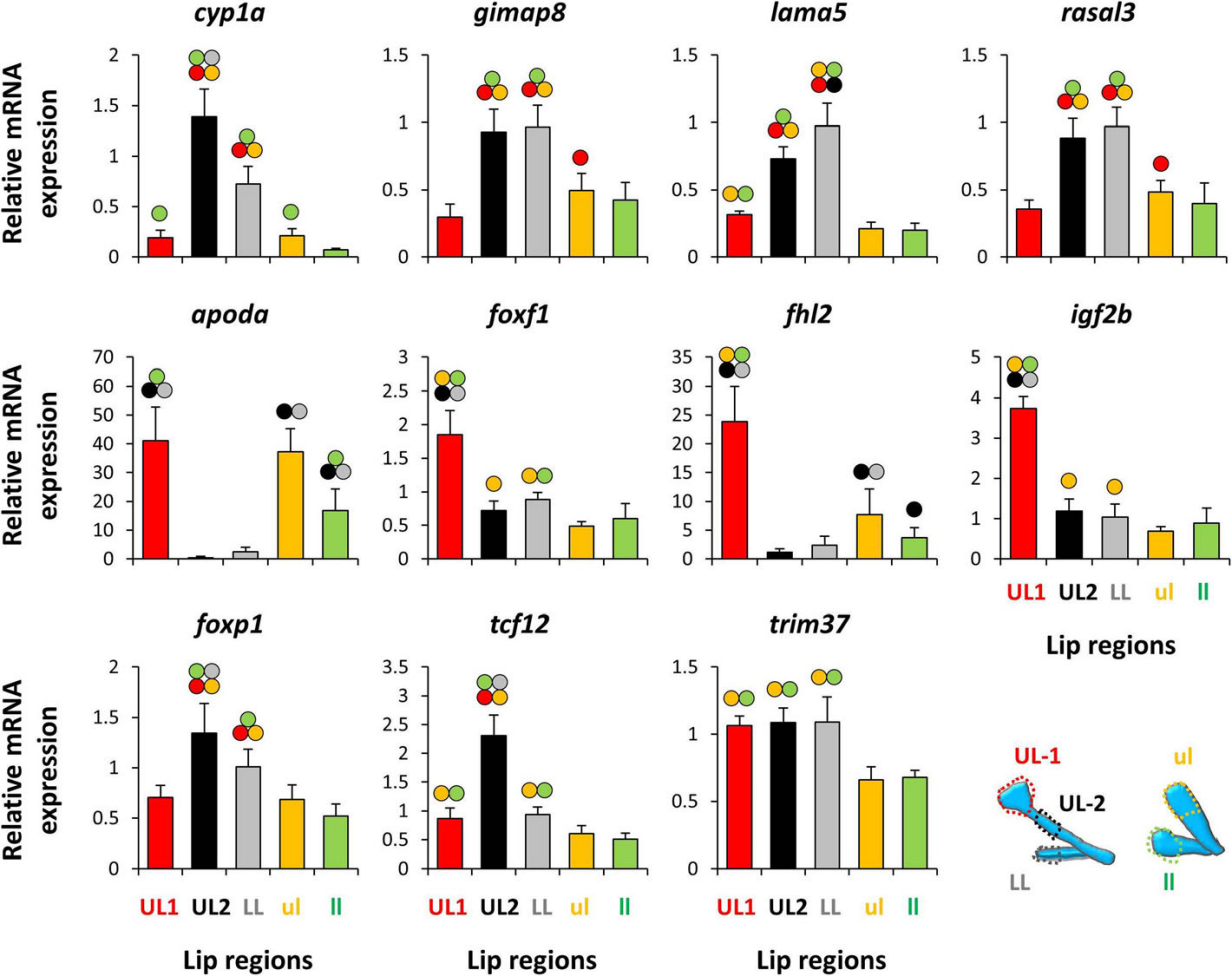
Expression analysis of a selection of candidate genes using qPCR. The bars represent means and standard deviations of RQ values for five biological replicates in each lip region. Circles above bars indicate significantly elevated expression (*P* < 0.05) in comparisons between the lip regions (i.e., compared to the bar matching the colour code of the circle)

Within the identified DE genes in the RNA-seq results, we selected nine genes with a known role in lip morphogenesis and other related functions in vertebrates (Table 3), as well as the two predicted upstream TFs (*foxp1* and *tcf12*) to be tested by qPCR (Fig. 5). Among these genes, *apoda*, *fhl2* and *gimap8*, were also implicated to be involved in lip hypertrophy in other cichlids (Table 3), and 5 genes, *apoda*, *cyp1a*, *lama5*, *rasal3* and *trim37* appeared to be differentially expressed in both intra- and inter-specific comparisons between hypertrophic and non-hypertrophic lips. Based on the RNA-seq results, four of the selected candidate genes have shown reduced expression in the hypertrophic UL-1 (*cyp1a*, *gimap8*, *lama5* and *rasal3*) whereas the other six genes (*apoda*, *foxf1*, *fhl2*, *igf2b* and *trim37*) had increased expression level in UL-1 of *G. permaxillaris*. The qPCR results showed that all of the genes followed similar expression patterns as found by RNA-seq, except for *trim37,* which showed no significant difference between the lip regions in *G. permaxillaris*. However, when the qPCR expression results for *G. permaxillaris* were compared to the two hypertrophic lip regions of *G. bellcrossi*, we found only *apoda* and *fhl2* showing increased expression in all the hypertrophic lip regions across both species. The four genes with reduced expression in UL-1 for *G. permaxillaris,* also showed reduced expression in the hypertrophic lip regions of *G. bellcrossi*. Among the two predicted TFs, only *foxp1* showed consistent difference between hypertrophic and non-hypertrophic lip regions across both species, with reduced expression in the hypertrophic lip regions. This indicates potential transcriptional repressor effects of *foxp1* on the downstream genes in the hypertrophic lip region. Altogether, the qPCR results show consistency with RNA-seq results, confirming the validity of our transcriptome data analysis in this study.

**Table 3:** Differentially expressed genes in the hypertrophic region of dorsal lip in *Gnathochromis permaxillaris* with related functions in vertebrates

Gene	Related function	Organism	References
*apoda*	A multi-ligand transporter involved in neural cell survival found to be repressed in lip tissue of thick-lipped Midas cichlid	Human Mouse Cichlid	(Dassati, Waldner & Schweigreiter, 2014) [30] (Manousaki et al., 2013) [7]
*cyp1a*	Involved in xenobiotic and steroid metabolism and associated with lip and palate cleft and cancers	Human	(Linnenkamp et al., 2020) [31] (Stuppia et al., 2011) [32]
*fhl2*	A transcriptional modulator of cell proliferation and apoptosis, found to be repressed in lip tissue of thick-lipped Midas cichlid	Human Cichlid	(Ng et al., 2011) [33] (Labalette et al., 2008) [34] (Manousaki et al., 2013) [7]
*foxf1*	Micro-deletion causes lip and palate deformities	Human Mouse	(Shaw-Smith, 2010) [35] (Xu et al., 2016) [36]
*foxp1*	Non-functional mutation causes pronounced vermilion border of upper lip	Human	(Meerschaut et al., 2017) [37]
*gimap8*	A GTP-binding protein with role in immunity and apoptosis, found to be repressed in lip tissue of thick-lipped Midas cichlid	Cichlid	(Manousaki et al., 2013) [7]
*igf2*	Expression changes in Russell–Silver syndrome leads to thin vermilion border of upper lip	Human	(Peñaherrera et al., 2010) [38]
*lama5*	An extracellular matrix glycoprotein mediating the attachment, migration and organization of cells into tissues, and implicated in lip inflammation and carcinogenesis	Human	(Peixoto da-Silva et al., 2012) [39]
*rasal3*	A negative regulator of Ras pathway and its duplication and deletion are both linked to defective lip morphogenesis	Human	(Draaken et al., 2013) [40] (Kosaki et al., 2011) [41]
*Trim37*	A tripartite motif family member involved in developmental patterning and its mutation causes lip and palate cleft	Human	(Kumpf et al., 2013) [42]
*tcf12*	Non-functional mutation causes thin upper lip and craniofacial deformities	Human	(Piard et al., 2015) [43]

## Discussion

Delineating the molecular basis of adaptive morphologies is essential to understand how they develop and evolve, and how they may contribute to adaptive radiation. In cichlids, lip hypertrophy is considered a craniofacial morphological novelty, which might promote incipient sympatric speciation through divergent selection (on feeding performance) and non-random/assortative mating [13, 15, 44]. The repeated evolution of hypertrophic lip phenotypes in cichlids inhabiting different lakes provides an opportunity to study the role of craniofacial soft tissues in adaptive radiations [8, 13, 44–46], as well as their underlying molecular mechanisms. There are only few studies that addressed this morphological novelty at molecular levels in cichlids [7, 8]. The lip hypertrophy phenotype is variable among cichlids, for example cichlid species studied so far show hypertrophy in both upper and lower lips [7, 8], while the selected target species in this study, *G. permaxillaris,* displays such a phenotype only in the anterior part of its upper/dorsal lip. This may indicate distinct molecular mechanisms involved in shaping seemingly similar craniofacial novelty across cichlids. In this study, we investigated the soft-tissue craniofacial trait of the hypertrophic lip in *G. permaxillaris,* in a comparative transcriptomic framework with *G. bellcrossi*, which are both from the Limnochromini tribe belonging to the cichlid adaptive radiation of Lake Tanganyika. *Gnathochromis permaxillaris* is a deepwater cichlid living over mud bottom, as far as oxygenated water reaches down, and has a unique mode of foraging.

Using differential gene expression analysis in the hypertrophic lip region as input for gene ontology analysis, we found multiple biological processes to be enriched. These processes involve cell motility, adhesion and developmental growth, as well as regulation of GTPase mediated signals, particularly, the Ras signalling pathway. A recent integrated genomic and transcriptomic study in humans has revealed that cell adhesion, cell junction and extracellular structure organizations are among the major biological processes involved in lip and cleft development and morphogenesis [47]. Furthermore, as studies on human and mouse have shown, biological processes involving developmental growth and cell proliferation are known to be the key mechanisms in upper lip development and morphogenesis [48]. On the other hand, RAS/MAPK signalling is among the well-known molecular pathways involved in development and morphogenesis of craniofacial structures in vertebrates [4, 49]. Activation of Ras signalling promotes cell proliferation, growth and survival in various tissues [50, 51], and because of these roles, several components of the Ras pathway are considered as therapeutic targets in different types of cancer [52]. In mammals, Ras signaling plays a pivotal role in skin development, dermal thickenning and skin carcinogenesis [53]. Defective activity of the Ras pathway can cause a wide range of skin anomalies, such as thickened palms and soles, redundant skin, papilloma formation, excessive proliferation of keratinocytes and increased skin folds [53]. We found components of Ras signaling to be differentially expressed including *rasal3,* an inhibitor of the pathway (discussed below), *rrad*, a direct downstream target of Ras signal [54], a scaffold protein involved in signal transduction *akap12b* [55]*,* and two genes encoding enzymes with roles in this signal; *arhgef18b* and *arhgef3l* [56, 57]. Therefore, the hypertophic lip region in the anterior upper lip of *G. permaxillaris* can be a result of increased activity of Ras signaling in this region. However, further functional studies (such as a protein manipulation method recently used in cichlids to investigate regional activity of a growth signal [10]) are required to find out the molecular reason for the anotomically limited activation of this signal only in the anterior part of the upper lip in *G. permaxillaris*. Interestingly, Ras signaling is also implicated among the pathways mediating molecular effects of environmentally induced mechanical stimuli in mammalian cells, raising the possiblity of its involvement in regulation of the plasticity of the lip phenotype [58].

We found that many of the genes with increased expression in the hypertrophic region of dorsal lip were already demonstrated to play a role in lip morphogensis and pathobiology in other vertebrates, including *alx1* [59], *alx3* [60], *angptl2* [61], *arid3a* [62], *crip2* [63], *ddr2* [64], *dpysl2* [65], *itga5* [66], *lmna* [67], *mgp* [68], *rgs5* [69], *rhob* [69, 70], *rxfp2* [71], *sostdc1* [69, 72], *thbs3* [73], *vcan* [74, 75], and *vtn* [76]. Among the transcriptionally repressed genes, we also found candidates with functions which were previously implicated in defective lip morphogenesis in other vertebarates such as *cd96* [77], *ep300* [78, 79], *fgfrl1* [80], *frzb* [81], *hectd1* [82, 83], *mmp13* [76], *slc16a6* [84], and *syne1* [85]. Since most of these studies were conducted on mammals, our results suggest that a similar set of genes might be involved in lip morphogenesis, not just in teleosts, but across vertebrates. Moreover, considering that all the previous studies in mammals linking these genes individually to lip morphogenesis, our findings for the first time show that these genes might be co-regulated or have regulatory interactions in an interconnected network. Thus, further functional studies are required to investigate their specific role in morphological divergence of soft tissues in fish.

Among the genes with reduced expression in the hypertophic lip regions, we validated four genes with qPCR, *cyp1a*, *gimap8*, *lama5* and *rasal3*, and found all of them to show a similar expression reduction pattern in all of the hypertophic lip regions of both species. Cytochrome P450 (CYP) 1 alpha, *cyp1a*, encodes an enzyme with an important role in the cytochrome p450 xenobiotic metabolism and the synthesis of steroids and other lipids. *Cyp1a* is a downstream target for AHR, RAS and Wnt/β-Catenin signaling pathways [86, 87], and all of these signals are demonstrated to play important roles in morphogenesis and adaptive radiations of craniofacial elements in teleost fishes [4]. Differential regulation of *cyp1a* is implicated in craniofacial morphological divergence in fish [88]. Differential regulation of AHR, RAS and Wnt/β-Catenin signals, as well as *cyp1a,* is also involved in the defective formation of the lip and palate in vertebrates [31, 32, 89–91]. We found reduced expression of *cyp1a* in the hypertophic lip regions in both cichlid species, which can be explained by its inhibitory role on cell proliferation [92, 93].

The second gene, GTPase immunity-associated protein family member 8 or *gimap8*, encodes a nucleotide-binding protein that plays a role in the maintenance and survival of lymphocytes in mammals [94]. Consistent with our result, the same gene was found to be repressed in lip tissue of thick-lipped Midas cichlid from Nicaragua [7]; however, its exact function during development and morphogenesis of soft tissues in vertebrates remained unclear. In humans, increased expression of *gimap8* orthologue has been reported during adipocyte differentiation, indicating its potential role in cell differentiation [95]. The third gene, *lama5*, encodes one of the vertebrate laminin alpha chain proteins, a family of extracellular matrix glycoproteins, which are the major noncollagenous constituents of basement membranes. In humans, *lama5* has been implicated in pathologenesis of lip inflammation and carcinogenesis [39]. In mouse, loss of *lama5* causes hyper-proliferation of basal keratinocytes, an increase in the number of immune cells and thickening of epidermis; thus, reduced expression of *lama5* in the hypertophic lip regions might result in an increased number of keratinocytes and subsequently thicker epidermis in these regions [96]. The last gene, *rasal3*, RAS protein activator like-3, encodes a negative regulator of Ras signalling pathway and its duplication and deletion are both linked to defective lip morphogenesis in humans [40, 41]. The reduced expression of *rasal3* in the hypertophic lip regions suggests higher activity of RAS signaling in this region, which is consistent with the enriched RAS related gene ontology for the differentially expressed genes.

Two of the genes with increased expression in hypertrophic lip regions, *apoda* and *fhl2*, were particularly interesting, since both genes have been already reported as potential molecular players in the formation of thick-lipped phenotype of Central American cichlids [7]. However, in contrast to our results, the expression of both genes have been shown to be repressed in hypertophic lips of Midas cichlid. Four-and-a-half LIM domains 2, *fhl2*, encodes a transcriptional modulator of cell proliferation [33, 34], while apolipoprotein Da, *apoda*, encodes a multi-ligand transporter involved in neural cell survival [30]. The molecular reason for this discrepancy is unclear, but it is likely that these genes have dual and opposite modulatory functions under different cellular conditions. Future functional investigations are required to unravel this discrepancy. It should be noted that *fhl2* is also reported as an important molecular player in the formation of egg-spot in cichlids, indicating its functional diversity in the adaptive morphological divergence of cichlid fishes [97].

We predicted binding sites for Forkhead Box (FOX) transcription factors and the basic helix-loop-helix (bHLH) for transcription factor 12 (tcf12/heb) to be enriched on upstream regulatory sequences of many of the differentially expressed genes by RNA-seq. Among the differentially expressed genes, we only found *foxf1* as a potential candidate that could bind to the enriched FOX binding site. Interestingly, micro-deletion in mammalian orthologue of *foxf1* have been shown to be associated with lip and palate deformities in mouse and human [35, 36]. However, the qPCR analysis revealed that, although *foxf1* expression was increased in the hypertophic lip region of *G. permaxillaris*, its expression was not increased in the hypertophic lip regions of *G. bellcrossi*. This could indicate that another member of the FOX family might be involved, whose expression difference was not detected by RNA-seq. In addition to the core FOX binding site, we also found a binding site for *foxp1* (another member of FOX family) to be enriched multipe times on the regulatory sequences. The qPCR analysis revealed that *foxp1* expression has a significant expression reduction in all the hypertophic lip regions of both species. The reduced expression of *foxp1* suggests a potential repressive effect on transcription of the genes induced in the hypertophic regions. Interestingly, *foxp1* has been already demonstrated to have repressive effects on transcription of many of its downstream targets [98–100]. Moreover, a mutation affecting *foxp1* function has been associated with hypertophy of vermilion borders (side edges) of upper lip in humans [37]. The role of *foxp1* in lip hypertophy might be linked to its function in inhibition of cell proliferation by repressing the transcription of growth/cell cycle stimulating factors [101–103]. On the other hand *foxp1* expression in different mammalian tissues seems to be affected by a variety of environmental stimuli such as hypoxia [104], noise [105], environmentally induced epigenetic changes [106] and mechanotransduction [107], raising the possiblity of its involvement in regulation of the plasticity of the lip phenotype [58]. The second predicted TF, *tcf12*, has been recently shown to be involved in hypertophy of frontal head soft tissues (nuchal hump) in another East African cichlid species [11], however, we did not find its consistent expression difference between the lip regions by neither the RNA-seq nor the qPCR method in this study.

## Conclusions

Understanding the molecular basis of morphological novelties is crucial to understand how they evolve and contribute to adaptation and speciation. Using the hypertrophic lip in the Lake Tanganyika endemic *G. permaxillaris*, we lay the foundation for studying locally restricted soft tissue morphogenesis in vertebrates. In this study, we found an interconnected gene regulatory network underlying the formation of locally restricted hypertophy of dorsal lip which is a rare phenotype observed in a cichlid fish, *G. permaxillaris*. We also found few shared differentially expressed genes that may play a role in lip hypertrophy across two closely related species: *G. permaxillaris* and *G. bellcrossi.* Future investigations, including more distantly related cichlid species are required to understand whether similar set of genes are involved in the formation of regional lip hypertrophy across cichlids from different continents and other teleost fishes. In the future, we also advocate the integration of gene regulatory network analyses from various craniofacial tissues to understand how they collectively govern trophic and behavioural adaptations during cichlid adaptive radiation.

## Methods

### Fish rearing and tissue sampling

Five captive bred males of *G. permaxillaris* and five males of *G. bellcrossi* were raised in a large tank (approximately 2000 l), together with same numbers of females per species, in an environment enriched with various stony shelters to minimize competition stress. Both species had the same age (young adult between 7 and 8 months) and displayed similar swimming behaviour, but different feeding behaviours, with little or no intra- or inter-species aggression. We fed both species with the same diet, which is adjusted for Tanganyika cichlids (Tropical Tanganyika multi-ingredient flakes suitable for omnivorous cichlids). We sampled the species at the same time, when the protrusion of the anterior part of upper lip in *G. permaxillaris* males had appeared, while both upper and lower lips of *G. bellcrossi* were thickened as well (Fig. 1). At this young adult stage, both species were displaying sexual behaviours, such as chasing females and territorial defending. Before the dissection step, the fish were placed in a solution with 0.2 g MS-222 per 1 L water, and after being sacrificed, the lip regions specified in Fig. 1, which include epidermis, dermis and the underlying soft connective tissues in those regions, were all dissected. The entire tissues for each lip region per fish were considered as one biological replicate and were placed into separate tubes containing RNAlater (Qiagen) and stored at −20 C°. The fish sacrifice was performed based on the guidelines issued by the Federal Ministry of Science, Research and Economy of Austria and according to regulations of BMWFW. The study was carried out in compliance with the ARRIVE guidelines.

### RNA extraction and cDNA synthesis

Total RNA was extracted from 15 lip tissue samples (five replicates per lip region) from *G. permaxillaris* and ten lip tissue samples from *G. bellcrossi* using the ReliaPrep™ RNA Tissue Miniprep System Kit (Promega). Each sample consisted of epidermis, dermis and the underlying soft connective tissues in the specified lip regions (Fig. 1). Tissue samples were placed into tubes containing 250 μl of lysis buffer mixed with 1-Thioglycerol and 1.4 mm ceramic beads. The tissues were homogenized thoroughly by FastPrep-24 Instrument (MP Biomedicals, CA, USA) and RNA extraction was performed based on manufacturer’s ReliaPrep™ protocol for fibrous tissues. The extraction protocol followed included a column-based genomic DNA removal step and several purification steps. The total RNAs were diluted in 50 μl nuclease-free water and were quantified by a Nanophotometer (IMPLEN GmbH, Munich, Germany). The qualities of total RNAs were measured by R6K ScreenTape System using an Agilent 2200 TapeStation (Agilent Technologies), and all samples had a RNA integrity number (RIN) minimum of eight. Around 200 ng of the extracted total RNA from each sample was used for cDNA synthesis based on the manufacturer’s protocol of High Capacity cDNA Reverse Transcription kit (Applied Biosystems) and 1:4 times cDNA dilution was used as input to perform qPCR.

### RNA-seq library, transcriptome assembly and gene expression

To obtain a list of gene transcripts from the lip tissues, we performed RNA-seq library preparation, based on the protocol of Standard TruSeq Stranded mRNA Sample Prep Kit (Illumina) with 500 ng of total RNA per tissue input. We assessed the library qualities by running them on a D1000 ScreenTape (Agilent) using TapeStation 2200 (Agilent). We diluted the libraries to an optimal quantity recommended for sequencing, and then pooled the libraries with equal molarity. To generate 125 bp paired-end reads, the RNA-sequencing was performed in the NGS Facility at Vienna Biocenter Core Facilities (VBCF, Austria) on an Illumina HiSeq2500. Next, the raw reads were de-multiplexed based on the unique barcodes incorporated in each sample during the library preparation step. The quality control step was conducted on the raw reads from each sample using the FastQC analysis tool [108]. For each sample, the low quality reads were discarded, following the standard quality trimming step of the Trimmomatic software [109]. To do this, the filtering criteria was scaled to only maintain the reads with phred +33 quality score of at least 34 for all bases for a minimum length of 50 bp. The de novo transcriptome assembly of the lip tissues was implemented using the quality trimmed paired-end reads of all samples through the Trinity package [110, 111].

The transcript abundances were calculated using the assembled transcriptome and Kallisto tool within the Trinity package, in order to attain the transcript expression levels in each sample [112]. Next, we conducted the conversion from transcript to gene level quantification of the abundances using RSEM software [113], which is a step bundled with Trinity package [110]. The gene expression levels were compared between the lip regions within each species. For each comparison, the transcripts abundances of all samples included in the comparison were used to build a normalized expression matrix by Trinity software. Subsequently, transcripts showing differential expression were identified through edgeR package [114–117] and the R Bioconductor software (R version 3.4.4, R Development Core Team 2018). We have used the TMM (trimmed mean of M-values) scaling normalization that aims to account for differences in total RNA production across all samples, which is recommended for data normalization across samples with variable read counts [115, 118]. The differentially expressed genes were filtered by a false-discovery rate (FDR) cutoff of 0.05 [119] and those with minimum of 2 fold-expression change were used to create heatmaps. We converted gene IDs of the differentially expressed genes to zebrafish orthologues gene IDs with well annotated signalling pathways and biological processes using the BioMart package [120]. Finally, the enrichment for gene ontology (GO) terms for biological process was performed through Database for Annotation, Visualization and Integrated Discovery (DAVID) [121]. Furthermore, the knowledge-based interactions between the gene products (109 DE genes) were investigated by STRING v10 (http://string-db.org/), using zebrafish databases for protein interactomes [122].

### Primer design and qPCR

The qPCR primers for candidate genes (selected based on the RNA-seq results), were designed after aligning their assembled sequences to their homologous sequences from other East African cichlid tribes from Lake Tanganyika [123–125], as well as *Oreochromis niloticus* (Tilapiini). Through these alignments, we identified the conserved sequence regions across East African cichlids at the exon junctions (using CLC Genomic Workbench, CLC Bio, Denmark, and annotated genome of *Astatotilapia burtoni* from the Ensembl database, http://www.ensembl.org). The primers were designed for short amplicon size (< 200 bp) using Primer Express 3.0 (Applied Biosystems, CA, USA), and their secondary structures and dimerization were assessed through OligoAnalyzer 3.1 (Integrated DNA Technology) (Table S2). The qPCR steps provided by the protocol of Maxima SYBR Green/ROX qPCR Master Mix (2X) (Thermo Fisher Scientific, Germany) were performed following the guidelines for optimal experimental set-up for each qPCR run [126]. The qPCR program was set for 2 min at 50 °C, 10 min at 95 °C, 40 cycles of 15 sec at 95 °C and 1 min at 60 °C, followed by an additional step of dissociation at 60 °C – 95 °C. The primer efficiency (E values) for each gene was calculated through standard curves generated by serial dilutions of pooled cDNA samples. The standard curves were run in triplicates and calculated using the following formula: E = 10[− 1/slope] (Table S2).

In order to select candidate reference genes, we used the transcriptome data and followed an approach that we have already used in our previous studies [11, 29, 127, 128]. In brief, we first identified the genes showing no expression difference (FDR = 1) between the lip regions for each species and ranked them according to their level of expression to attain those with highest expression. Next, we ranked the genes based on their coefficient of variation (CV of expression levels) across the replicates and we selected the top ten genes shared between the transcriptome comparisons of both species as candidate reference genes. Finally, after qPCR expression analysis of the ten genes across all samples, we ranked them based on their expression stability by three different algorithms: BestKeeper [129], NormFinder [130] and geNorm [131]. Thus, we used the geometric means of the Cq values of the top two most stable reference genes to normalize Cq values of target genes in each sample (ΔCq target = Cq target – Cq reference). The relative expression levels (RQ) were calculated by 2^−ΔΔCq^ approach [132] and the log-transformed RQ values were used for ANOVA and Tukey’s HSD post hoc tests to calculate statistical differences between the groups.

## Supplementary Information

**Additional file 1: Table S1.** Number of RNA sequencing reads obtained for each sample and differentially expressed genes identified in the RNA-Seq experiment.

**Additional file 2: Table S2.** qPCR primers for candidate reference and target genes.

## Data Availability

All the data represented in this study are provided within the main manuscript or in the supplementary materials. The raw sequence reads have been submitted to the Sequencing Read Archive (SRA) of NCBI with accession number: PRJNA694145 (Permanent link: www.ncbi.nlm.nih.gov/bioproject/PRJNA694145/).
